# Detection of Human Papillomavirus in Squamous Lesions of the Conjunctiva Using RNA and DNA In-Situ Hybridization

**DOI:** 10.3390/ijms23137249

**Published:** 2022-06-29

**Authors:** Cornelia Peterson, Rupin N. Parikh, Meleha T. Ahmad, Ashley A. Campbell, Yassine Daoud, Nicholas Mahoney, Sepideh Siadati, Charles G. Eberhart

**Affiliations:** 1 Department of Molecular and Comparative Pathobiology, The Johns Hopkins University School of Medicine, Baltimore, MD 21205, USA; cpeter52@jhmi.edu; 2 Department of Ophthalmology, The Johns Hopkins University School of Medicine, Baltimore, MD 21287, USA; rparikh91@gmail.com (R.N.P.); meleha.ahmad@gmail.com (M.T.A.); acampb23@jhmi.edu (A.A.C.); ydaoud1@jhmi.edu (Y.D.); nick.mahoney@jhmi.edu (N.M.); 3 Department of Pathology, The Johns Hopkins University School of Medicine, Baltimore, MD 21231, USA; ssiadat1@jhmi.edu

**Keywords:** human papillomavirus, conjunctiva, papilloma, conjunctival intraepithelial neoplasia, carcinoma in situ, squamous cell carcinoma, in-situ hybridization

## Abstract

In-situ hybridization provides a convenient and reliable method to detect human papillomavirus (HPV) infection in formalin-fixed paraffin-embedded tissue. Cases of conjunctival papillomas, conjunctival intraepithelial neoplasia (CIN), conjunctival carcinoma in situ (cCIS), and invasive squamous cell carcinoma (SCC), in which low-risk (LR) and/or high-risk (HR) HPV types were evaluated by RNA or DNA in-situ hybridization, were retrospectively identified. LR HPV types were frequently detected in conjunctival papillomas (25/30, 83%), including 17/18 (94%) with RNA probes, compared to 8/12 (75%) with DNA probes. None of the CIN/cCIS or SCC cases were positive for LR HPV by either method. HR HPV was detected by RNA in-situ hybridization in 1/16 (6%) of CIN/cCIS cases and 2/4 (50%) of SCC cases, while DNA in-situ hybridization failed to detect HPV infection in any of the CIN/cCIS lesions. Reactive atypia and dysplasia observed in papillomas was generally associated with the detection of LR HPV types. Collectively, our findings indicate RNA in-situ hybridization may provide a high-sensitivity approach for identifying HPV infection in squamous lesions of the conjunctiva and facilitate the distinction between reactive atypia and true dysplasia. There was no clear association between HPV infection and atopy in papillomas or dysplastic lesions.

## 1. Introduction

The ocular surface barrier, predominately comprising the conjunctival epithelium, is uniquely vulnerable to chronic environmental exposure and microbial infection [1,2]. As a result, numerous etiologies have been proposed for the spectrum of squamous conjunctival proliferations, which range from benign papillomas to dysplastic lesions. The latter are also referred to as ocular surface squamous neoplasms (OSSNs) and include pre-invasive conjunctival intraepithelial neoplasia (CIN) and conjunctival carcinoma in situ (cCIS), in addition to invasive squamous cell carcinomas (SCC) [3,4,5,6,7,8,9]. While complete surgical excision with a no-touch technique is generally curative, when margins are positive, recurrence can occur, and medical therapies are frequently now incorporated into treatment [10]. Chronic exposure to ultraviolet radiation (UVR) is commonly implicated in both premalignant and malignant OSSNs due to deleterious effects on the DNA damage repair system, while the epitheliotropic and oncogenic human papillomavirus (HPV) is frequently detected in conjunctival papillomas [9,11,12,13,14,15,16,17,18,19,20,21].

Atopy has also been reported as a risk factor in OSSN development. Several proposed mechanisms for atopy-associated tumorigenesis have been hypothesized, including pruritus-induced epithelial microtrauma with secondary HPV infection, type I hypersensitivity-mediated inflammation and tissue remodeling, and competitive binding of IgE receptors by allergen-specific IgE, limiting anti-tumor immune responses [22,23,24,25,26]. Indeed, HPV infection is the most well documented of these proposed etiologic contributors, and as discussed below, a range of studies has investigated the potential role of HPV in OSSN.

It has been estimated that as many as 400 types of HPV, a small, spherical, non-enveloped, double-stranded DNA virus, exist, and approximately 200 have been fully characterized, with mucosal types grouped into low- (LR HPV) and high-risk (HR HPV) subtypes based on potential for malignant transformation [13,22,27,28,29,30,31,32,33]. HPV types 6 and 11 are categorized as LR HPV and are most commonly associated with conjunctival papillomas, while HR HPV 16 and 18, among others, have been implicated as contributing factors in the development of some conjunctival papillomas and OSSNs [1,9,12,13,14,19,22,31,34,35,36,37,38,39,40]. HR HPV DNA integrates into the host genome to induce expression of viral genes E6 and E7 to disrupt p53 and pRB, respectively, effectively propagating and dysregulating the cell cycle [31,41,42].

A number of studies suggest HPV, alone, is sufficient as a causative agent for conjunctival lesions; however, others propose a potential two-hit etiology, requiring prior UVR damage, chemical mutagenesis, or immunosuppression in addition to HPV infection [1,5,12,13,16,18,20,22,31,35,43,44,45]. Considerable variability in the correlation of HPV infection with squamous lesions has been reported, and this may be due to the variety of methods for both sampling and viral detection [31,35,46,47]. Immunohistochemical approaches, including direct detection of HPV-encoded proteins or alterations in p16 expression, have been investigated, but more recent studies suggest that these methods are prone to false-positive results in conjunctival lesions [13,22,34,41,46,48,49,50,51,52,53,54,55,56,57,58,59]. Historically, HPV DNA was detected with Southern blotting or in-situ hybridization assays, which can have poor sensitivity, and polymerase chain reactions (PCR), with high false-positive rates [31,47]. More recently, RNA-based methodologies, such as real-time PCR (RT-PCR) and in-situ hybridization for HPV mRNA, have become more reliable alternatives, particularly for squamous lesions of the head and neck [31,47,60].

RNA in-situ hybridization permits direct visualization of intralesional transcriptionally active virus, with probes capable of detecting E6 and E7 mRNA, and provides morphologic evaluation of nuclear HPV localization within virally infected neoplastic cells [51,52,60,61,62,63,64,65,66,67]. In addition to the reported high sensitivity and specificity, viral mRNA in-situ hybridization continues to gain popularity due to the automated staining platform, which facilitates rapid turnaround time and standardization across diagnostic laboratories and incorporation into standard slide-based workflows [60,61,62,65,68,69,70,71]. Here, we present a case series of in-situ hybridization for detection of LR and HR HPV in conjunctival papillomas and OSSNs with the objective of describing the detection of HPV infection in these lesions, using either DNA or RNA approaches, and determining the degree to which HPV is associated with atopy. The study period included a transition in our pathology laboratory from DNA to RNA in-situ hybridization, and cases assessed using either method are included. Lesions were evaluated by DNA in-situ hybridization prior to 2015 and RNA in-situ hybridization from 2015 onward, based solely on the availability of testing offered by the clinical laboratory at the time of initial case evaluation.

## 2. Results

### 2.1. Clinicopathologic Characteristics

HPV in-situ hybridization was performed on 53 lesions, including 33 papillomas, 16 CIN/cCIS, and 4 invasive SCCs. Demographic and clinicopathologic information for these patients is provided in Table 1, with complete information for individual cases presented in Table S1.

**Figure 1:**
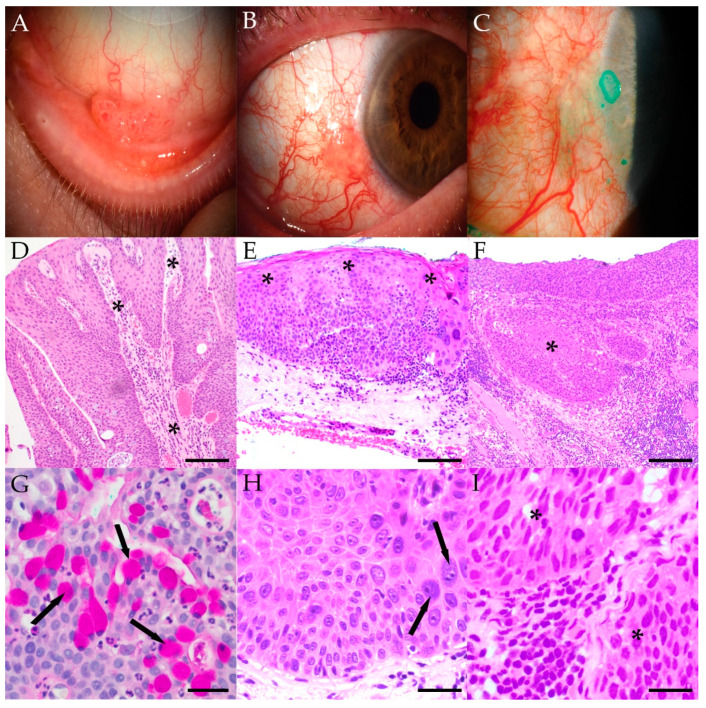
Clinical appearance of squamous bulbar conjunctival lesions including a lobulated papilloma extending toward the inferior fornix from Case 3 (**A**), a more nodular CIN encroaching on the limbus from Case 39 (**B**), and a larger invasive perilimbal SCC with prominent conjunctival hyperemia from Case 51 (**C**). Representative low-magnification histology of conjunctival lesions including a papilloma characterized by hyperplastic epithelium overlying fibrovascular core denoted with asterisks (**D**) (scale bar: 100 μM), a CIN exhibiting a thickened and dysplastic epithelium (asterisks) (**E**) (scale bar: 50 μM), and an invasive SCC with neoplastic cells breaching the epithelial basement membrane and infiltrating the substantia propria (asterisk) (**F**) (scale bar: 100 μM). Higher magnification of a representative conjunctival papilloma with large numbers of PAS-positive goblet cells (arrows) and infiltrating neutrophils (**G**) (scale bar: 25 μM), CIN with abnormal epithelial stratification and hyperchromatic and pleomorphic nuclei (arrows) (**H**) (scale bar: 25 μM), and invasive SCC with epithelial dysplasia (asterisks) and intervening inflamed stroma (**I**) (scale bar: 25 μM).

Representative clinical photographs and photomicrographs of each type of lesion are presented in Figure 1. Conjunctival papillomas were generally rich in goblet cells, with epithelium covering fibrovascular cores. Cases of CIN and CIS featured dysplastic cells involving the conjunctival epithelium to varying extents, with sharply defined peripheral edges, but no invasion of underlying stroma. Superficial keratinization was variable, and in some cases, with more prominent thickening of the epithelium, sessile papillomatous changes were noted. Invasive SCC featured nests and strands of tumor cells in the substantia propria. No goblet cells, mucinous, or glandular components were identified in the CIN, CIS, or SCC cases reported here.

### 2.2. In-Situ Hybridization

In-situ hybridization using DNA probes was performed in 14/33 (42%) of papillomatous lesions and 4/16 (25%) CIN/cCIS lesions. In-situ hybridization using RNA probes was performed in the remaining lesions: (19/33 (58%) papillomas, 12/16 (75%) CIN/cCIS, and 3/3 invasive SCCs). Low-risk types were detected in 25/30 (83%) papillomas, 0/11 CIN/cCIS, and 0/1 invasive SCCs. High-risk types were detected in 2/28 (7%) papillomas, 1/16 (6%) CIN/cCIS, and 2/4 (50%) invasive SCCs. Detection of LR and HR HPV types was mutually exclusive in papillomatous lesions, and four lesions (12%) were negative for both LR and HR HPV.

HPV signal presented in epithelial cells as dot-like foci visible at 100× magnification in the nucleus. Multiple dots in at least a subset of cells were required to call a case positive. In some cases, the small intracellular signals were quite numerous, and could become almost confluent within a single nucleus. Staining was restricted to epithelial cells, and stromal elements, including blood vessels and mesenchymal cells, were negative in all cases examined. In cases containing normal conjunctiva adjacent to a lesion, the non-neoplastic epithelium was also always negative.

**Figure 2:**
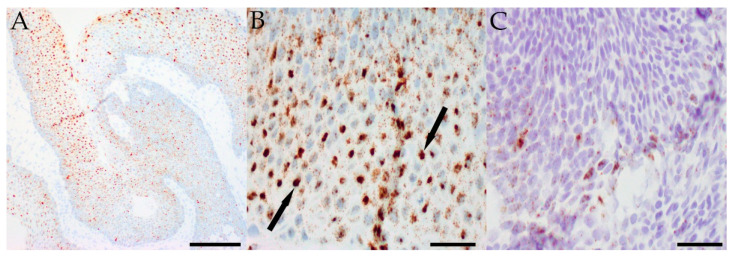
Representative images of in-situ hybridization showing diffuse low-risk HPV positivity in a papilloma from Case 6 using RNA probes (**A**) (scale bar: 100 μM). At higher magnification, many nuclei are almost completely filled with smaller dot-like foci of staining (arrows) (**B**) (scale bar: 25 μM). More focal high-risk HPV positivity in a papilloma from Case 16 using DNA probes (**C**) (scale bar: 25 μM).

**Figure 3:**
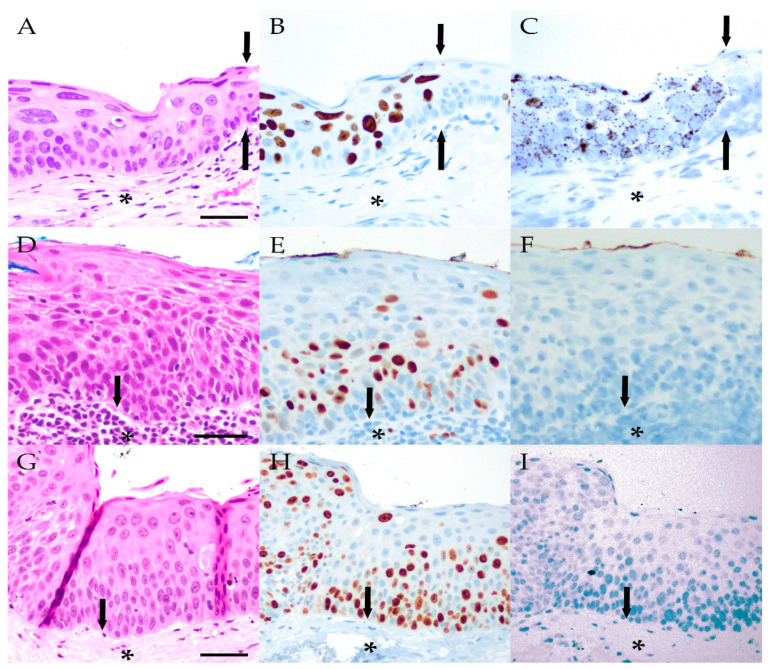
Representative images from the periphery of an H&E-stained CIN from Case 39 showing the transition to non-neoplastic conjunctiva (**A**) (scale bar: 25 μM). Increased nuclear immunolabeling for Ki67 within the neoplastic epithelium (**B**) and corresponding RNA in-situ hybridization demonstrating positivity for high-risk HPV within the same dysplastic cells (**C**). Arrows in A–C mark the border between CIN and adjacent conjunctiva with non-dysplastic epithelium on the right side of each panel negative for Ki67 and HPV. The underlying substantia propria, containing blood vessels and inflammatory cells, is marked by asterisks and is negative for HPV. Representative images of an H&E-stained CIN from Case 40 (**D**) (scale bar: 25 μM) and cCIS from Case 44 (**G**) (scale bar: 25 μM) with arrows pointing to the epithelial base and asterisks in the underlying substantia propria. Increased nuclear immunolabeling for Ki67 within the neoplastic epithelium (**E**,**H**) and the corresponding RNA in-situ hybridization, which were both negative for high-risk HPV (**F**,**I**).

With respect to the extent of staining within individual lesions, HPV was present diffusely in most papillomas. Focal HPV positivity was observed in 8/25 (32%) LR HPV-positive papillomas and 1/2 (50%) HR HPV-positive papillomas, while diffuse staining was noted in 17/25 (68%) and 1/2 (50%) LR and HR HPV-positive papillomas, respectively. In contrast, HPV expression was more localized in the OSSN cases that contained virus. Focal HPV positivity was observed in the single HR HPV-positive CIN (Case 39) and both HR HPV-positive invasive SCC lesions. Further details of HPV in-situ hybridization are summarized in Table 2, and representative images for positive LR and HR HPV in-situ hybridization are presented in Figure 2 and Figure 3.

### 2.3. Correlation of HPV with Histologic Features

**Figure 4:**
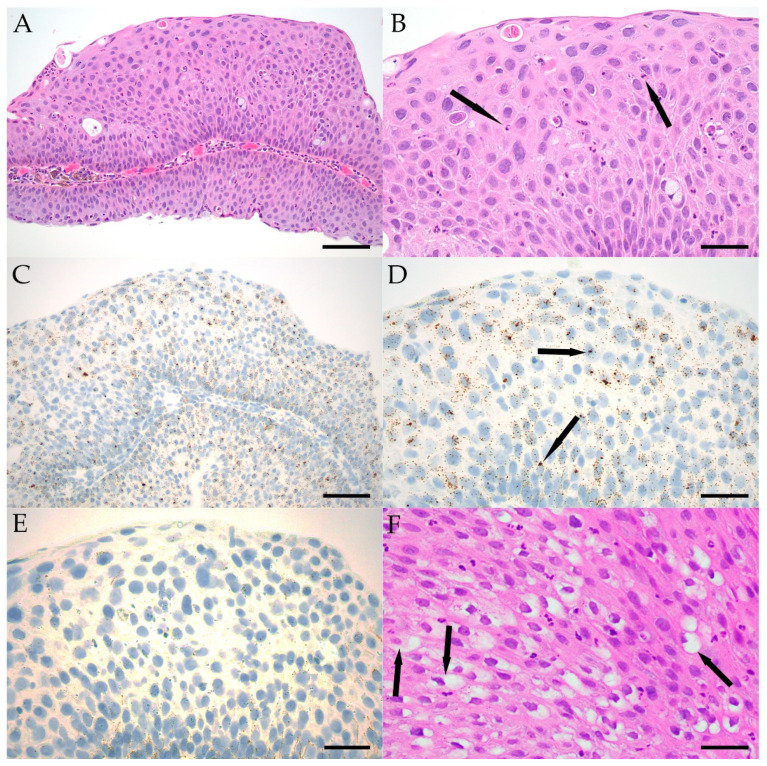
H&E-stained section of Case 1, a conjunctival papilloma demonstrating reactive epithelial atypia associated with inflammation (**A**) (scale bar: 50 μM). Reactive atypia and mixed inflammation characterized by neutrophils (arrows) and fewer lymphocytes and plasma cells within the epithelium from Case 1 at higher magnification (**B**) (scale bar: 25 μM). HPV in Case 1 was confirmed with RNA in-situ hybridization, with diffuse LR HPV in (**C**) (scale bar: 50 μM) and shown at higher magnification with dot-like foci of signal, some of which coalesced into larger masses (arrows) (**D**) (scale bar: 25 μM). HR HPV was not detected in these same epithelial cells (**E**) (scale bar: 25 μM). Diffuse koilocytosis (arrows) in the well-differentiated epithelium of a recurrent LR HPV-positive papilloma from Case 14 (**F**) (scale bar: 25 μM).

Conjunctival papillomas can become quite inflamed, leading to reactive epithelial atypia, in which epithelial cells show generally mild nuclear enlargement and pleomorphism, along with some chromatin changes and more prominent nucleoli. Reactive atypia can sometimes be difficult to distinguish from dysplasia, although the latter process is often less uniform and more severe. Detection of LR HPV could potentially be helpful in such cases and support a less aggressive biological potential if positive. In 18 of 33 (55%) papilloma cases, intraepithelial inflammation and epithelial atypia diagnosed as reactive were detected. Dysplasia was rarer, and noted in 4/25 (16%) LR HPV-positive papillomas, 1/2 (50%) HR-positive papillomas (Case 29), and none of the HPV-negative papillomas. In three LR HPV-positive papillomas diagnosed with dysplasia, the focal in-situ hybridization signal corresponded to the foci of dysplastic epithelium, while in Case 33 and Case 39, discrete foci of epithelial dysplasia were observed, but more diffuse LR and HR HPV signal was noted. An inflamed papilloma with reactive atypia and LR HPV-positive epithelium is demonstrated in Figure 4A–E.

Koilocytic cells were identified in all lesion types but were relatively rare, with focal change observed in 2/6 (33%) of HPV-negative papillomas, 10/25 (40%) LR HPV-positive papillomas, 1/15 (7%) HPV-negative CIN/cCIS, and 1/2 (50%) HR HPV-positive invasive SCCs. More diffuse koilocytic-appearing cells were observed in 1/25 (4%) LR HPV-positive papillomas, as shown in Figure 4F. Immunolabeling for p16 was performed on seven papillomas, five CIN/cCIS, and three invasive SCCs at the time of initial case evaluation. Generally, p16 labeling corresponded to HPV detection. However, p16 labeling was observed in two CIN/cCIS and one SCC in which HPV was not detected.

### 2.4. Patient Follow-Up and Atopic History

Patients returned for follow-up care in 17/33 (52%) papilloma cases, 8/16 (50%) CIN/cCIS cases, and none of the invasive SCCs. The length of follow-up (mean ± SD) was 605 ± 1219 days and 887 ± 1534 days for papilloma and CIN/cCIS cases, respectively. Recurrence was noted in only one case (Case 14) following an initial diagnosis of an HPV-negative papilloma. The recurrent lesion was originally treated with excision and subsequently observed.

History of atopic disease, including asthma, eczema, or allergic rhinitis or conjunctivitis, was also recorded for patients when possible. Electronic medical records were available for review for 18/33 (56%) of all papillomas, including 3 HPV-negative lesions, 14 LR HPV-positive lesions, and 1 HR HPV-positive lesion. Atopic disease, including asthma (1/3), rhinitis/conjunctivitis (1/3), and a combination of the two (1/3), was reported in patients with HPV-negative papillomas. A history of asthma was present in 9/14 (64%) patients with LR HPV-positive papillomas, 2 of whom also had additional atopic disease, including eczema and rhinitis/conjunctivitis, respectively. Two additional patients with LR HPV-positive papillomas had histories of eczema and rhinitis/conjunctivitis, alone. There was no reported history of atopic disease in the single HR HPV-positive papilloma. For cases of CIN/cCIS, there was no history of atopic disease in the HR HPV-positive lesion that had medical records available for review.

## 3. Discussion

The prevalence of HPV infection in conjunctival epithelial proliferations has been difficult to establish, due, at least in part, to the range of detection methods used [35,72]. To some degree, this has been dependent on the type of lesion, and findings in conjunctival papillomas have been more uniform over time [14,31,36]. Our results using DNA and RNA in-situ hybridization were similar to previous studies demonstrating LR HPV infection in the majority of conjunctival papillomas using PCR, as shown in Table 3 [12,13,14,18,31,36,37,44]. In the current study, LR HPV was detected in 94% of papillomas using RNA probes, while only 75% were positive for LR HPV when evaluated by DNA probes. Interestingly, prior studies using DNA probes had lower rates of HPV detected, perhaps reflecting the age of the studies [73]. While some single cases reports examined papillomas using RNA in-situ hybridization, this study represents the first series of cases, and the larger percentage of papillomas found to be positive using RNA in-situ hybridization could potentially reflect the increased sensitivity of this approach [74,75]. However, given the relatively small number of cases in each group, the differences between DNA and RNA in-situ approaches could also be due to normal variation. The cases that were negative for HPV by DNA in-situ hybridization were all several years old, and because of the known potential for RNA degradation, these were not retested using RNA in-situ hybridization.

Conjunctival papillomas can occasionally become inflamed and show prominent reactive atypia, which can be difficult to distinguish from clinically worrisome dysplasia. Among the 33 papillomas we examined, generally mild dysplasia was identified in 5 cases (15%). This number may be elevated due to selection bias, as papillomas with concerning microscopic features were more likely to have had HPV status analyzed during the study period. LR HPV was detected in four of five potentially dysplastic papillomas, while HR HPV was only detected in one. This suggests that the majority of conjunctival papillomas with atypia or dysplasia are associated with LR HPV, and that in-situ analysis could be useful in supporting limited malignant potential in many of these cases. Similar to these findings at the ocular surface, LR HPV was detected in the majority of papillomas, including those with atypical features of the nasopharynx, oral cavity, and larynx, while HR types are more commonly detected in invasive SCCs of these tissues [78,79,80,81,82,83,84].

The reported prevalence of HPV in CIN and other more aggressive OSSN is more variable, with positive cases almost always containing high-risk types [31,34,35,85]. In prior studies, 40–100% of CIN/cCIS were HPV-positive by DNA in-situ hybridization and 0–31% positive by RNA in-situ hybridization [22,34,46,86]. Prevalence was higher in SCC (30–36% positive by DNA in-situ hybridization and 16–26% positive by RNA in-situ hybridization) [34,46,85,86]. In our cohort, 1 CIN/cCIS out of 12 (8%) was positive for HR HPV using RNA in-situ hybridization, while none of the 4 cases evaluated by DNA in-situ hybridization were positive, suggesting that RNA approaches may provide higher sensitivity for detection. The overall positive rate for the 16 cases examined was, thus, 6%.

Immunohistochemical approaches, including direct detection of HPV-encoded proteins or alterations in p16 expression, have been investigated, but more recent studies suggest that these methods are prone to false-positive results [13,22,41,46,49,51,53,55,56,58,59,82]. In particular, it has been found that many lesions negative for HPV express p16 [49,58]. Next-generation sequencing represents another potential method by which HPV would be detected in routine clinical samples [87,88,89]. With respect to clinical advances in care, imaging, including optical coherence tomography, is increasingly being used to distinguish benign and malignant ocular surface lesions [10,87,88,89,90].

Many demographic and clinical associations in our study were similar to prior reports. Papillomas of the conjunctiva reportedly occur more commonly in younger patients, while more dysplastic OSSN lesions tend to present in older ones, and our cases had a mean age of 43, 68, and 74 years at the time of presentation for papillomas, CIN/cCIS, and invasive SCCs, respectively [3,40,77,87]. Prior studies suggested that conjunctival papillomas and OSSN are more common in male patients, and this was true overall in our group as well, except for the relatively small set of SCC [3,7,21,40,46]. Papillomas in our cohort presented more frequently on palpebral conjunctiva (78%), while CIN/cCIS cases were more common for bulbar conjunctiva (82%). However, while a recent report suggested correlation of HR HPV-positive OSSN with atopy, our study did not identify a history of atopic disease in patients with HR HPV lesions [22]. Another group reported increased keratinization in HPV-negative OSSN, but we did not identify a similar association in our cases [46].

Limitations of the current study include the relatively small number of invasive SCC cases, precluding the possibility of statistical comparisons between groups. In addition, detailed review of electronic medical records was not possible for all cases in this series, as many were older pathology consultations submitted by outside physicians. While RNA in archived formalin-fixed paraffin-embedded tissue is labile and sensitive to degradation, potentially affecting the sensitivity of in-situ hybridization, we do not believe this is of concern in our study, as all in-situ hybridization assays were performed around the time of initial histologic review [63,65].

## 4. Materials and Methods

### 4.1. Study Design

A retrospective review of the Ophthalmic Pathology Archives of The Johns Hopkins Hospital for cases matching keyword searches including “conjunctiva,” “in-situ hybridization,” and “human papillomavirus” between 2007–2021 was conducted. Electronic medical records were reviewed for the following clinical and pathologic information: age at presentation, gender, lesion type and location, clinical treatment modalities, length of clinical follow-up, history of recurrence, history of atopic disease, HPV in-situ hybridization assay details, and details regarding any additional immunohistochemical stains. Any case for which in-situ hybridization was performed to detect LR and/or HR HPV in a conjunctival papilloma, CIN/cCIS, or invasive squamous cell carcinoma was included. Cases were reviewed to confirm the initial diagnosis, and intralesional koilocytic or dysplastic features were noted.

### 4.2. HPV In-Situ Hybridization

HPV in-situ hybridization was performed by the Johns Hopkins Hospital clinical laboratories at the time of initial review of histology using standard protocols. Lesions assayed prior to 2015 were subject to DNA ISH (Ventana Medical Systems; Tucson, AZ, USA), and those evaluated from 2015 and onward were analyzed using RNA probes (Advanced Cell Diagnostics Inc; Hayward, CA, USA). Both DNA and RNA probes for LR HPV detect the same two types (6, 11), while DNA probes for HR HPV detect 12 types (16, 18, 31, 33, 35, 39, 45, 51, 52, 56, 58, and 68) and RNA probes detect 18 HR HPV types (16, 18, 26, 31, 33, 35, 39, 45, 51, 52, 53, 56, 58, 59, 66, 68, 73, and 82). While 37/53 lesions (70%) were evaluated for both LR and HR types, evaluation by either DNA or RNA approaches was mutually exclusive. The commercially available probes utilized in this study were designed to hybridize with E6/E7 in the various HPV types. All determinations of in-situ hybridization positivity were performed by an ocular pathologist (CGE).

## 5. Conclusions

We confirmed that LR HPV is frequently detected in conjunctival papillomas while HR HPV is rarely detected in these lesions. The fact that most papillomas with histopathological dysplasia were positive for LR HPV suggests that their malignant potential is limited, and that in-situ hybridization may help with prognostic assessment, although analysis of more cases with long-term follow-up will be needed to confirm this. HR HPV was detected by RNA in-situ hybridization in a relatively small number of CIN and SCC, but was not clearly linked to atopy or other clinical and histopathological features.

## Figures and Tables

**Table 1:** Clinicopathologic Characteristics of Cases of Conjunctival Papillomas and OSSNs.

Lesion Type	Cases	Age Mean ± SD (Range)	Sex N (%)	Bulbar (%)	Palpebral (%)	Forniceal (%)
**Papilloma**	33	43 ± 17 (9–86)	Male 18 (54) Female 15 (46)	4/27 (15)	21/27 (78)	2/27 (7)
**CIN/cCIS**	16	68 ± 10 (49–90)	Male 11 (69) Female 5 (31)	9/11 (82)	2/11 (18)	0/11
**SCC**	4	74 ± 12 (61–90)	Male 1 (25)	0/1	1/1	0/1
			Female 3 (75)			
**Total**	53	53 ± 20 (9–90)	Male 30 (56) Female 23 (44)	13/39 (33)	24/39 (62)	2/39 (5)

CIN: conjunctiva intraepithelial neoplasia, cCIS: conjunctival carcinoma in situ, SCC: invasive squamous cell carcinoma.

**Table 2:** HPV Detection in Conjunctival Papillomas and OSSNs.

		Cases	Low-Risk Types Positive	High-Risk Types Positive
RNA ISH	Papilloma	19	17/18 (94%)	1/15 (7%)
	CIN/cCIS	12	0/8	1/12 (8%)
	SCC	4	0/1	2/4 (50%)
	Papilloma	14	8/12 (75%)	1/13 (8%)
DNA ISH	CIN/cCIS	4	0/3	0/4
	SCC	0	--	--
Overall	Papilloma	33	25/30 (83%)	2/28 (7%)
	CIN/cCIS	16	0/11	1/16 (6%)
	SCC	4	0/1	2/4 (50%)

ISH: in-situ hybridization, CIN: conjunctiva intraepithelial neoplasia, cCIS: conjunctival carcinoma in situ, SCC: invasive squamous cell carcinoma.

**Table 3:** HPV Detection in Conjunctival Papillomas Reported in the Literature.

HPV Prevalence	Method of Detection	Low-Risk Types Positive	High-Risk Types Positive	Reference
0/14	PCR	0/14	0/14	[13]
86/106 (81%)	PCR	85/86 (99%)	1/86 (1%)	[14]
19/25 (76%)	PCR	19/19	--	[36]
	ISH (DNA)	9/19 (47%)	--	
4/4	PCR	4/4	0/4	[76]
48/52 (92%)	PCR	41/47 (87%)	1/47 (2%)	[77]
4/7 (57%)	PCR	3/4 (75%)	0/4	[73]
	ISH (DNA)	1/4 (25%)	0/4	

PCR: polymerase chain reaction, ISH: in-situ hybridization.

## Data Availability

Not applicable.

## Supplementary Materials

The following supporting information can be downloaded at: https://www.mdpi.com/article/10.3390/ijms23137249/s1.
