# Supplementary material for: Detection of Human Papillomavirus in Squamous Lesions of the Conjunctiva Using RNA and DNA In-Situ Hybridization
Source: Int J Mol Sci. 2022 Jun 29;23(13):7249. doi: 10.3390/ijms23137249 (PMC9266440; doi:10.3390/ijms23137249)
Supplement: Supplementary file 1 [file ijms-23-07249-s001.zip › Peterson et al IJMS HPV OSSN Supplemental Table 1.pdf]

Table S1. Clinicopathologic characteristics of individual cases of squamous conjunctival proliferations.

Lesion Type	Case #	Age (y)	Sex	Clinical Location of Lesion	LR-HPV Status	HR-HPV Status	Atopic Status
**Papilloma**							
	**1**	46	F	--	+	-	--
	**2**	38	M	--	+	-	--
	**3**	44	F	forniceal	+	-	asthma, rhinitis/conjunctivitis
	**4**	35	F	palpebral	+	-	rhinitis/conjunctivitis
	**5**	46	M	palpebral	+	-	asthma
	**6**	36	F	palpebral	+	-	asthma, eczema
	**7**	35	M	bulbar	+	-	--
	**8**	43	M	palpebral	+	-	none
	**9**	54	F	palpebral	+	-	--
	**10**	47	F	palpebral	+	NP	none
	**11**	9	F	palpebral	-	-	rhinitis/conjunctivitis
	**12**	11	F	forniceal	-	-	asthma
	**13**	38	M	palpebral	+	-	none
	**14**	56	M	palpebral*	+	-	asthma
	**15**	32	F	palpebral	+	-	asthma
	**16**	31	M	palpebral	NP	+	none
	**17**	57	M	palpebral	+	-	--
	**18**	43	M	--	NP	-	--
	**19**	86	M	bulbar	-	-	--
	**20**	33	M	palpebral	+	-	eczema
	**21**	48	M	--	+	-	--
	**22**	70	F	bulbar	-	-	--
	**23**	24	F	bulbar	+	-	asthma
	**24**	13	M	palpebral	+	NP	asthma
	**25**	24	F	palpebral	+	-	--
	**26**	64	F	--	+	-	--
	**27**	37	M	palpebral	+	-	--
	**28**	55	F	palpebral	-	NP	asthma, rhinitis/conjunctivitis
	**29**	60	F	palpebral	NP	+	--
	**30**	49	M	palpebral	+	-	asthma
	**31**	37	M	palpebral	+	-	--
	**32**	35	M	palpebral	+	NP	asthma
	**33**	67	M	forniceal	+	-	--
**CIN**							
	**34**	80	M	bulbar	-	-	--
	**35**	72	F	bulbar	-	-	--
	**36**	74	M	--	NP	-	--
	**37**	63	M	bulbar	-	-	--
	**38**	53	M	palpebral	-	-	--
	**39**	49	M	bulbar	-	+	none
	**40**	72	M	bulbar	NP	-	asthma
	**41**	83	F	palpebral	-	-	none
	**42**	73	M	--	-	-	--
**cCIS**							
	**43**	89	M	bulbar	NP	-	none
	**44**	69	M	--	-	-	--
	**45**	71	M	--	-	-	--
	**46**	61	F	--	NP	-	--
	**47**	56	F	bulbar	-	-	--
	**48**	69	F	bulbar	-	-	--
	**49**	61	M	bulbar	NP	-	none

**SCC**							
	**50**	72	F	--	-	+	--
	**51**	61	F	--	NP	-	--
	**52**	90	M	palpebral	NP	+	--
	**53**	73	F	--	NP	-	--

--: case submitted as outside consult; EMR unavailable , CIN: conjunctiva intraepithelial neoplasia, cCIS: conjunctival carcinoma *in situ*, SCC: invasive squamous cell carcinoma, NP: not performed; *: recurrent lesion
